# Trap Modulated Charge Carrier Transport in Polyethylene/Graphene Nanocomposites

**DOI:** 10.1038/s41598-017-04196-5

**Published:** 2017-06-21

**Authors:** Zhonglei Li, Boxue Du, Chenlei Han, Hang Xu

**Affiliations:** Key Laboratory of Smart Grid of Education Ministry, School of Electrical and Information Engineering, Tianjin University, Tianjin, 300072 China

## Abstract

The role of trap characteristics in modulating charge transport properties is attracting much attentions in electrical and electronic engineering, which has an important effect on the electrical properties of dielectrics. This paper focuses on the electrical properties of Low-density Polyethylene (LDPE)/graphene nanocomposites (NCs), as well as the corresponding trap level characteristics. The dc conductivity, breakdown strength and space charge behaviors of NCs with the filler content of 0 wt%, 0.005 wt%, 0.01 wt%, 0.1 wt% and 0.5 wt% are studied, and their trap level distributions are characterized by isothermal discharge current (IDC) tests. The experimental results show that the 0.005 wt% LDPE/graphene NCs have a lower dc conductivity, a higher breakdown strength and a much smaller amount of space charge accumulation than the neat LDPE. It is indicated that the graphene addition with a filler content of 0.005 wt% introduces large quantities of deep carrier traps that reduce charge carrier mobility and result in the homocharge accumulation near the electrodes. The deep trap modulated charge carrier transport attributes to reduce the dc conductivity, suppress the injection of space charges into polymer bulks and enhance the breakdown strength, which is of great significance in improving electrical properties of polymer dielectrics.

## Introduction

Nanodielectric materials have been one of the research hotspots in electrical and electronic engineering, since its concept was first proposed by Lewis in 1994^1^. During the past two decades, plenty of research has been done on preparation, evaluation, and characterization of novel nanocomposites (NCs), and most of the results presented that the NCs showed better electrical, thermal and mechanical performances than the original polymers and microcomposites^2–8^. Especially, their excellent electrical properties, including low permittivity, low conductivity, high breakdown strength as well as enhanced partial discharge (PD) and tracking resistance, make them the third generation of insulating materials^9–11^. The novel properties of NCs are thought to be ascribed to the small-scale effect, boundary effect and quantum size effect of nanoparticles that involve their high specific surface area^12^. In 2004 and 2005, Lewis and Tanaka published two research papers, giving deep insight into the microscopic interfaces between nanofillers and polymer matrix and trying to reveal the mechanism of the polymer-nanofiller interaction zones on the electrical performance^13, 14^. However, this mechanism is still incomplete now. Especially, the understanding of the correlativity among the microscopic interaction zones, the charge carrier transport in the mesoscopic view and the macroscopic electrical properties is not fully clear. Further investigation should be carried out to clarify the relationship among the microscopic, mesoscopic and macroscopic properties of NCs.

In previous investigations, various nanofillers, including oxides (e.g. aluminium oxide, silicon oxide, titanium oxide, magnesium oxide and zinc oxide), nitrides (e.g. aluminium nitride and boron nitride), montmorillonite (MMT) clay and so on, were doped into polymers to prepare NCs^15–19^. Additionally, with the development of organic chemistry synthesis, the surface modification of nanofillers, such as surface grafting, were employed to achieve a better dispersion in polymer matrix, thus enhancing the effect of polymer-filler interaction zones^20, 21^. Nevertheless, it is difficult for the common nanofillers mentioned above to achieve a specific surface area of 1000 m^2^/g in theory^22, 23^. Monolayer graphene, as a unique nano-scaled filler with a thickness of only an atomic layer, has an enormous specific surface area up to ~2000 m^2^/g, which may significantly increase the polymer-filler interaction zones and advantage to exploit the further potentials of nanodielectrics^24–27^.

Graphene was first discovered and characterized in 2004^28^. Its high mechanical strength, excellent flexibility, unusual optical properties as well as superior electrical and thermal conductivity make it promising for applications in infrared detectors, solar cells, light-emitting diodes (LED), quantum devices and so on^29, 30^. In the research field of insulation dielectrics, previous researches mainly focused on the electrical, thermal and mechanical properties of various polymer/graphene NCs in the near-range or above the percolation threshold. Gaska found that the LDPE composites containing 1 wt% graphene nanosheets showed non-linear electrical conductivities and enhanced mechanical properties^27^. Fim reported that the PE/graphene composites became more thermally stable, and semiconductive when the fill content was above a critical percolation threshold of ~3.8 vol%^31^. However, few study on the electrical properties of NCs with the very low filler content (far below the percolation threshold) has been carried out until now, and the mechanism of the graphene addition on the electrical properties of composites is still unknown.

In this research, Low-density Polyethylene (LDPE) is employed as polymer matrix, which is widely used as insulating material in electrical engineering. LDPE/graphene NCs with the filler content of 0 wt%, 0.005 wt%, 0.01 wt%, 0.1 wt% and 0.5 wt% are prepared by melt blending. The electrical properties of NCs, including the dc conductivity, breakdown strength and space charge behaviors, are measured. Meanwhile, their trap level distributions are characterized by isothermal discharge current (IDC) tests. Based on the results, a schematic model is proposed for illustrating the trap modulated charge carrier transport in LDPE/graphene NCs, thereby further revealing the relationship between the trap level distributions and the electrical properties.

## Results and Discussion

### Characterization of LDPE/graphene NCs

**Figure 1:**
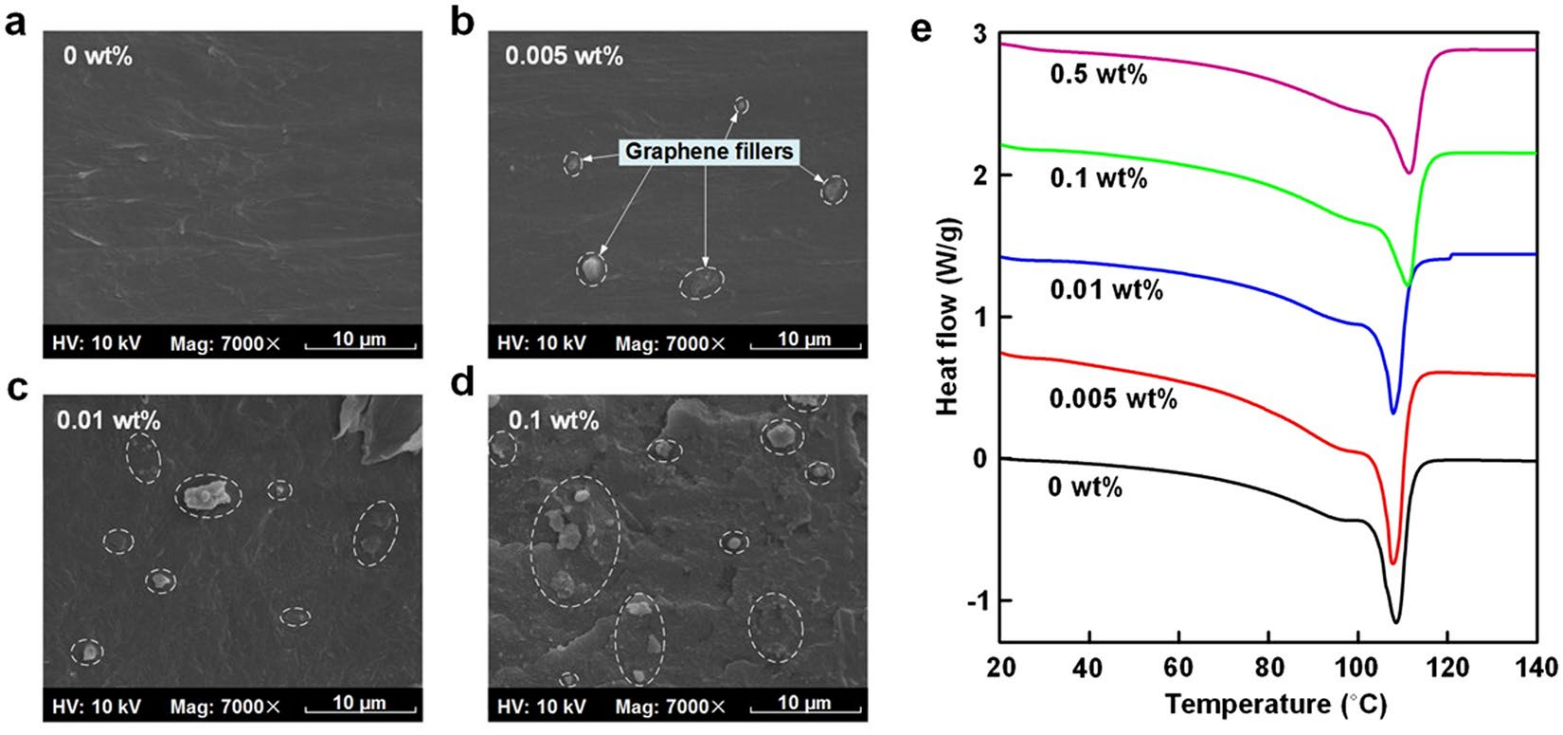
(**a**,**b**,**c** and **d**) are the SEM images of cross sections, corresponding to the 0 wt%, 0.005 wt%, 0.01 wt% and 0.1 wt% LDPE/graphene NCs respectively. (**e**) DSC curves of LDPE/graphene NCs as a function of filler content.

Figure 1a–d show the scanning electron microscope (SEM) images of cross sections, corresponding to the 0 wt%, 0.005 wt%, 0.01 wt% and 0.1 wt% LDPE/graphene NCs respectively. It can be observed that the graphene fillers are well-dispersed in the polymer matrix when the filler content is 0.005 wt% and 0.01 wt%. With the increase of filler content, the volume fraction of the graphene fillers goes on rising, and the nearest distance between neighbor fillers (NDNF) decreases gradually. It is noted that in the SEM image of 0.1 wt% NCs, the NDNF becomes at the level of sub-micrometer or nanometer.

Figure 1e presents the differential scanning calorimeter (DSC) curves of LDPE/graphene NCs. Through the integral of DSC curves, the crystallinity (*X*) can be calculated by the formula as follows^32^:

1 $$X=\frac{{\rm{\Delta }}{H}_{m}}{{\rm{\Delta }}{H}_{0}}\times 100 \% $$

where ∆*H*_0_ is the melting enthalpy of LDPE fully crystallized (100%) and generally ∆*H*_0_ = 293 J/g^33^. ∆*H*_*m*_ is the melting enthalpy of LDPE NCs investigated. The melting temperature and melting enthalpy are shown in Table 1. The degree of crystallinity characterized by DSC tests is 32.6%, 39.5%, 37.0%, 34.6% and 32.5%, for 0 wt%, 0.005 wt%, 0.01 wt%, 0.1 wt% and 0.5 wt% LDPE/graphene NCs, respectively. It is indicated that the crystallinity at 0.005 wt% is highest, which is likely due to the heterogeneous nucleation. That is, the well-dispersed graphene fillers act as the heterogeneous nucleating agents, accelerate the rate of crystallization and enhance the crystallinity of LDPE composites. Nevertheless, when the filler content exceeds 0.01 wt%, the graphene fillers with a high aspect ratio could limit the movement of polymer molecular chains and leave little space for additional crystallization, thus leading to a decrease of crystallinity. It is also shown that the melting peak temperature slightly increases when the filler content exceeds 0.1 wt%.

**Table 1:** Melting temperature, melting enthalpy and crystallinity levels of LDPE/graphene NCs measured by DSC tests.

Specimens	Melting temperature (°C)	Melting enthalpy (J · g^−1^)	Crystallinity (%)
0 wt%	106.0	95.62	32.6
0.005 wt%	105.5	115.71	39.5
0.01 wt%	105.3	108.34	37.0
0.1 wt%	108.5	101.40	34.6
0.5 wt%	108.8	95.14	32.5

### DC conductivity and breakdown strength

**Figure 2:**
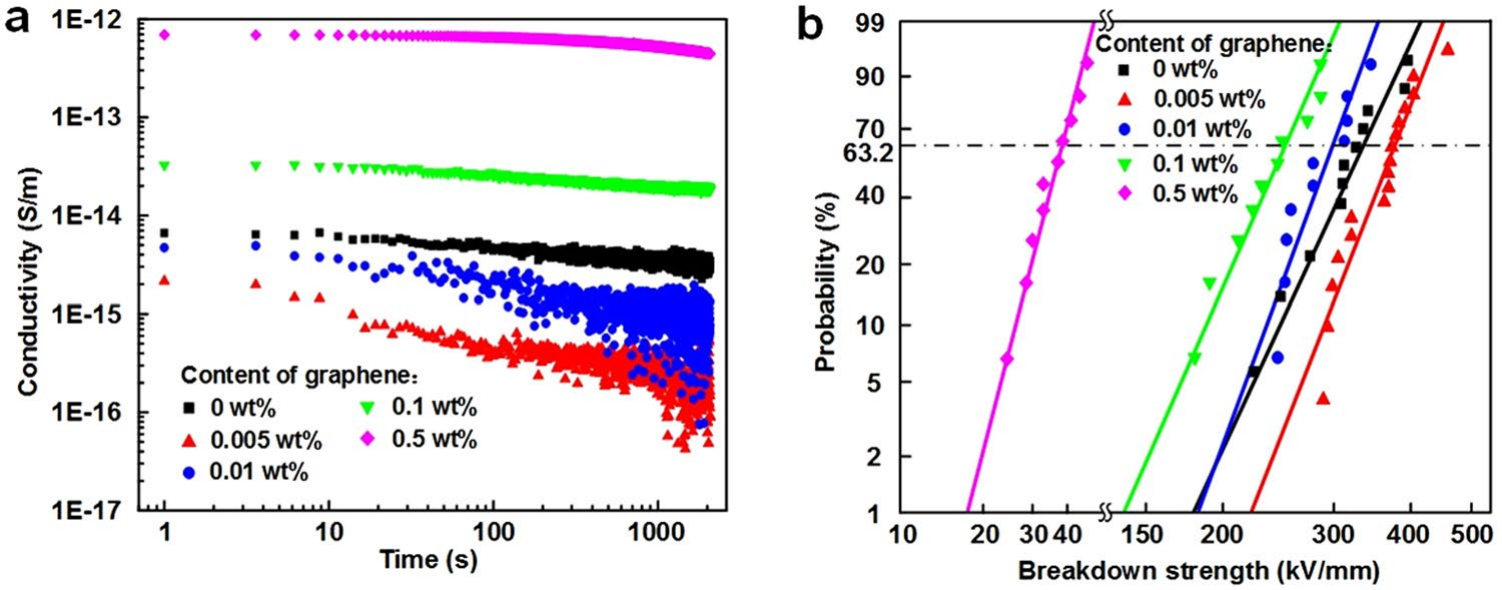
(**a**) DC conductivity of LDPE/graphene NCs as a function of polarization time under 10 kV/mm. (**b**) Weibull distribution of dc breakdown strength of LDPE/graphene NCs.

Figure 2a shows the dc conductivity of LDPE/graphene NCs as a function of polarization time under 10 kV/mm. It is observed that the conductivity of NCs decreases with the polarization time. Especially for the 0.005 wt% and 0.01 wt% specimens, there is a marked drop in the charging current during the first 100 s. After 100 s, the charging currents reach the quasi-steady state and decay at a slower rate. This is because the charging current is composed of a polarization current and a conduction current. With the lapse of polarization time, the polarization comes into stable state gradually and the conduction current becomes dominant^34^. However, for the 0.5 wt% LDPE/graphene composites, the charging current decays slowly during the measuring time and still does not reach a steady state up to 2000 s, which is ascribed to the injection and accumulation of space charge in the bulk rather to a slow polarization process^35^.

It is assumed in this study that the mean value of conductivity at the last 100 s represents the dc conductivity, which is 3.18 × 10^−15^ S/m, 1.98 × 10^−16^ S/m, 7.57 × 10^−16^ S/m, 2.02 × 10^−14^ S/m and 4.87 × 10^−13^ S/m for 0 wt%, 0.005 wt%, 0.01 wt%, 0.1 wt% and 0.5 wt% LDPE/graphene composites, respectively. The 0.005 wt% NCs has a significantly lower dc conductivity than the neat LDPE. It is known that as a zero-gap semiconductor, the graphene has a high conductivity under electric stress. Based on the percolation theory, when the filler content is 0.005 wt%, the fillers are well-dispersed in the polymer matrix and the NDNF extends to tens micrometers as shown in Fig. 1b, indicating the filler content of 0.005 wt% is much lower than the percolation threshold^36^. Therefore, it is speculated that no conducting path of charge carriers forms throughout the 0.005 wt% LDPE/graphene NCs even under high electric field. On the other hand, there are a large amount of interaction zones at the microcosmic interfaces between graphene fillers and LDPE matrix, attributed to the large specific surface area of graphene (1217 m^2^/g in this study). It is believed that the polymer-filler interaction zones can capture charge carriers, thus suppressing the transport of charge carriers. Adding up these microscopic effects, a macroscopic decrease of dc conductivity occurs.

Figure 2a also shows that, with the further increase of the filler content from 0.005 wt% to 0.1 wt%, the dc conductivity presents an increasing trend. As shown in Fig. 1a–d, the NDNF decreases gradually with the increase of filler content, and the NDNF in 0.1 wt% NCs becomes at the level of sub-micrometer or nanometer. As a result, the polymer-filler interaction zones may overlap with each other, which provides low-resistance paths for electrons and holes and accelerates a local transport of charge carriers, thus resulting in an increase of dc conductivity^37^. With further increasing the content to 0.5 wt%, the filler content is above a percolation threshold, leading to the thermally-activated field-enhanced carrier hopping at the polymer-filler interfaces under applied stress. In this case, a large number of charge carriers will pass through the thin polymer layer between neighboring fillers, thereby resulting in a dramatic rise of dc conductivity as shown in Fig. 2a ^36^.

Figure 2b demonstrates the Weibull distribution of dc breakdown strength of LDPE/graphene composites. The characteristic breakdown strength of 0 wt%, 0.005 wt%, 0.01 wt%, 0.1 wt% and 0.5 wt% LDPE/graphene NCs is 336.3 kV/mm, 379.4 kV/mm, 301.3 kV/mm, 252.9 kV/mm and 38.6 kV/mm, respectively. It is indicated that the breakdown strength is associated with the dc conductivity shown in Fig. 2a. That is, the specimen with a lower dc conductivity has a higher dc breakdown strength. The largest breakdown strength of 0.005 wt% LDPE/graphene composites is also attributed to its inhibiting effect of the polymer-filler interaction zones on the transport of charge carriers. With further increasing the filler content from 0.005 wt% to 0.5 wt%, the transport of charge carriers though the amorphous regions is accelerated, involving the trapping and detrapping processes. Li also found that the NCs with small quantities of nano-Al_2_O_3_ showed a lower electrical conduction and the improvement of breakdown properties^38^. It is known that the trapping and recombination of charge carriers would produce hot electrons due to non-radiative transition of energy via an Auger-type process^39^. The hot electrons may have sufficient energy to collide with a molecule and dissociate into free radicals. According to the theory proposed by Kao, this process can be expressed as ref. 40.

2 $$\begin{array}{rcl}{\rm{AB}}+{{\rm{e}}}^{-}({\rm{hot}}) & \to  & {{\rm{A}}}^{\ast }+{{\rm{B}}}^{\ast }+{{\rm{e}}}^{-}({\rm{cold}})\\ or & \to  & {{\rm{A}}}^{\ast }+{{\rm{B}}}^{\ast }+{{\rm{e}}}^{-}({\rm{trapped}})+{\rm{energy}}\,{\rm{release}}\end{array}$$

This process is continuous, resulting in a formation of low-density regions in the amorphous area, in which electrons are more easily to be accelerated by electric field and gain kinetic energies to cause chain scission. Consequently, the chain scissions or microvoids cause partial discharge and further develop into breakdown of polymer.

### Space charge behaviors

**Figure 3:**
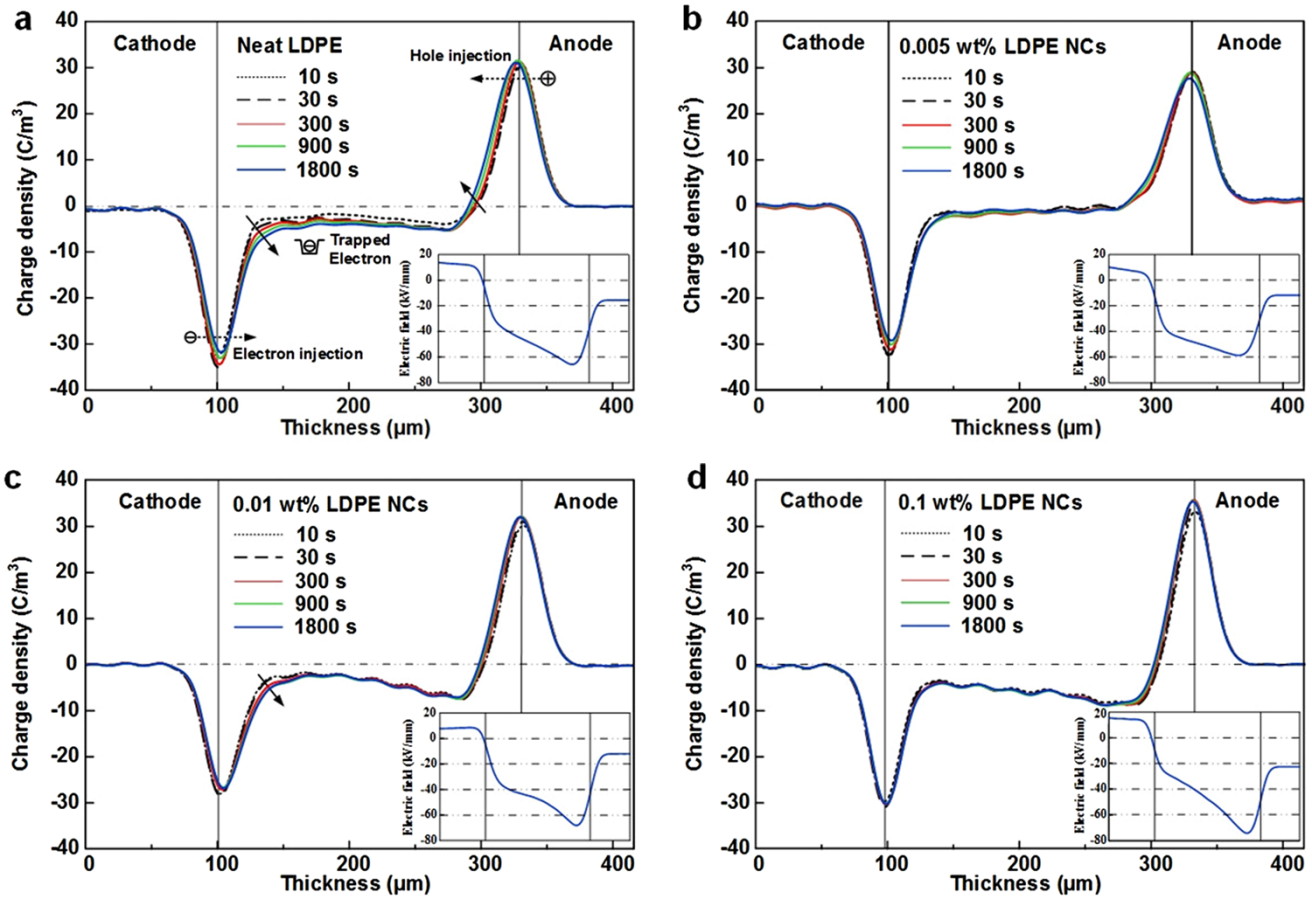
Space charge behaviors LDPE/graphene NCs under 50 kV/mm measured by PEA method, as well as the electric field distribution after applying the stress for 1800 s. (**a**) Neat LDPE. (**b**) 0.005 wt% LDPE NCs. (**c**) 0.01 wt% LDPE NCs. (**d**) 0.1 wt% LDPE NCs.

Figure 3 shows the dynamic space charge distributions of LDPE/graphene composites with the filler content of 0 wt%, 0.005 wt%, 0.01 wt% and 0.1 wt% under a dc electric field of 50 kV/mm for 1800 s. In addition, the electric field distributions after applying the stress for 1800 s are also presented in the bottom right corner, respectively. The space charge behaviors of neat LDPE are shown in Fig. 3a. It can be seen that both the charge peaks at electrode-polymer interfaces move towards the bulk of polymer with the lapse of polarization time, indicating a marked injection of homocharges from both of the electrodes. Due to the semicrystalline nature of LDPE, the interfaces between the crystalline and amorphous phases can introduce trapping sites, which would capture the injected charges and result in the accumulation of space charge. It is shown that a large quantity of electrons are injected deeper and trapped in the bulk of LDPE up to 1800 s, which is due to a much higher mobility of electrons than holes. Lewis has proved that holes in LDPE will tunnel between chain-located valence states through interchain barriers and electrons between chain states through chain barriers. As a result, a marked distortion of electric field occurs in the vicinity of anode.

By comparing the space charge behaviors in Fig. 3a and b, it is found that much fewer space charges accumulate in the bulk of 0.005 wt% LDPE NCs. According to the above-mentioned analyses, the polymer-graphene interaction zones suppress the charge carrier transport, leading to the large quantities of homocharges accumulating in the vicinity of both electrodes. It is believed that, the homocharges near the electrodes reduce the effective field at the electrode-polymer interface, enhance the potential barrier for Schottky injection, and suppress the further injection of homocharges from electrodes into polymer. With the lapse of polarization time, the space charge density in the bulk remain fairly stable, resulting in a uniform field-strength distribution. Up to 1800 s, the maximum electric field in the vicinity of anode is 58.6 kV/mm, which is much lower than that of neat LDPE (65.9 kV/mm).

However, further increasing the filler content would lead to an aggravation of space charge accumulation. As shown in Fig. 3c and d, large quantities of electrons are injected into the polymer bulk of 0.01 wt% and 0.1 wt% LDPE NCs. Combining with the results in Fig. 2a, it is assumed that the overlapping sites of the polymer-filler interaction zones would provide low-resistance paths for electrons between molecular chains, thus accelerating the transport of electrons through chain barriers and leading to significant amount of space charge accumulation in the bulk of polymer. Consequently, severe field distortions occur in the vicinity of anode as shown in Fig. 3c and d.

### Trap modulated charge carrier transport

**Figure 4:**
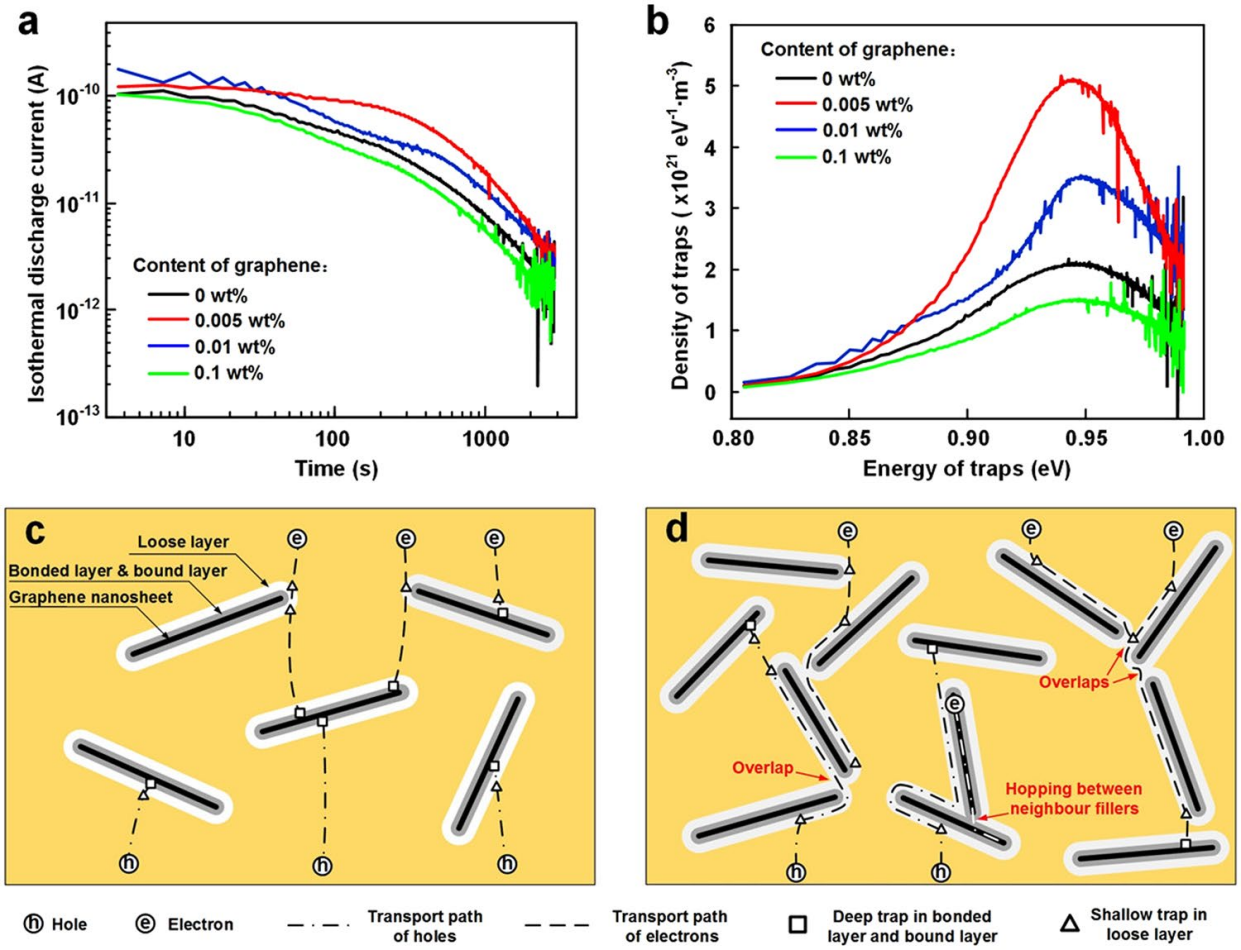
(**a**) Isothermal discharge current of LDPE/graphene NCs as a function of depolarization time. (**b**) Trap level distributions of LDPE/graphene NCs. (**c**) Schematic model for illustrating the deep trap modulated charge carrier transport. (**d**) Schematic model for illustrating the charge carrier transport through the overlaps of interaction zones and the thermally-activated field-enhanced hopping between neighbor fillers.

The experimental results presented above consistently show that the transport of charge carriers in 0.005 wt% LDPE/graphene NCs is significantly suppressed, resulting in a lower dc conductivity, a higher breakdown strength and a smaller amount of space charge accumulation than the neat LDPE. It is believed that the transport of charge carriers is closely associated with the trap level distributions in polymer. In this study, isothermal discharge current (IDC) tests are employed to characterize the trap level distributions of LDPE/graphene NCs. Figure 4a shows the isothermal discharge current (*I*) of NCs as a function of depolarization time (*t*). It is found that the discharge current decreases gradually with the depolarization time and finally reach a quasi-steady state, since deeper and deeper traps are involved in the charge-release mechanism. Based on the theory of IDC proposed by Simmons, the density of traps (*N*_*t*_(*E*)) and the energy of traps (*E*_*t*_) can be calculated by following equations^41^.

3 $${N}_{t}(E)=\frac{2dIt}{e{l}^{2}kT}$$

4 $${E}_{t}=kT\,\mathrm{ln}(\nu t)$$

where *d* is the thickness of the film and *l* is the penetration depth of injected electrons. *e* is the electronic charge quantity. *k* is the Boltzmann constant. *T* is the absolute temperature (323 K). *ν* is the escape frequency of trapped electrons, which is approximately equal to 10^12^ s^−1^ in LDPE.

The trap level distributions of LDPE/graphene NCs are obtained and shown in Fig. 4b. It is observed that the deep traps of all the LDPE/graphene NCs locate at ~0.95 eV. The density of deep traps increases with increasing filler content from 0 to 0.005 wt%, and then decreases with a further increase of filler content to 0.1 wt%. Previous researches have reported that the deep charge traps were introduced in NCs, such as aluminium oxide-doped epoxy (EP/Al_2_O_3_)^15^, silica-doped silicone rubber (SiR/SiO_2_)^16^ and titanium oxide-doped polyimide (PI/TiO_2_) NCs^17^. Tanaka proposed a multi-core model for typical spherical nanoparticles, in which the polymer-graphene interface consists of a bonded layer (the first layer), a bound layer (the second layer), a loose layer (the third layer) and an electric double layer overlapping the above three layers^14^. A large quantity of deep traps for electrons and holes are distributed in the bonded and bound layer, which capture charges within polymer and increase the average hopping distance for the charge carriers, thus suppressing the transport of charge carriers. As illustrated in Fig. 4c and d, a plate-like interaction zones appears at the interfaces between graphene fillers and LDPE matrix, referring to the multi-core model proposed by Tanaka. The inner layer, with a thickness of approximate ten nanometers, consists of the bonded layer and bound layer of the multi-core model, in which a large quantities of the deep carrier traps are distributed. The outer layer, with a thickness of tens of nanometers, presents the loose layer, which contains many shallow traps.

In the case of low filler content as shown in Fig. 4c, large quantities of deep carrier traps, introduced by the well-dispersed fillers, interact with the original trapping sites in LDPE matrix and result in a dramatic increase of deep trap density in NCs. Actually, the graphene fillers with a high aspect ratio has a large specific surface area of 1217 m^2^/g in this study, which is much larger than those of the spherical nanoparticles, such as Al_2_O_3_, SiO_2_ and TiO_2_. That is, graphene fillers provide much more deep traps than the other nanoparticles under the condition of same mass fraction. The large numbers of deep traps in the plate-like regions would capture holes and electrons and significantly suppress the transport of charge carriers in the microview, leading to the decrease of volume conductivity. As shown in Fig. 3b, the deep trap modulated carrier trapping results in the homocharges accumulation in the vicinity of electrodes, thus enhancing the potential barrier for Schottky injection, enhancing the electric field required for charge injection and suppressing the accumulation of space charges in the bulk. Additionally, the reduction of the injected charges could suppress the occurrence of charge trapping or recombination and reduce the formation of hot electrons with high energies that impact with molecules. Consequently, the breakdown strength is improved, based on the Kao’s model presented in Equation (2). Therefore, the deep trap modulated charge carrier transport in 0.005 wt% LDPE/Graphene composites leads to a lower dc conductivity, a higher breakdown and a smaller amount of space charge accumulation than the neat LDPE, as shown in Figs 2 and 3.

With the further increase of the filler content to 0.1 wt%, the NDNF becomes at the level of sub-micrometer or nanometer, as shown in Fig. 1d. Therefore, the loose layers would overlap with each other, which provides low-resistance paths for electrons and holes as illustrated in Fig. 4d. Besides, at a location where the polymer layer between the neighbor filler is thin enough, typical within tens of nanometers, a large amount of charge carriers may pass through the thin polymer layer between neighboring fillers via the thermally-activated field-enhanced hopping. The behaviors mentioned above would weaken the effects of deep traps in interaction zones and accelerate the local transport of charge carriers, thus further resulting in an increase of dc conductivity. Additionally, the effect of the deep traps on the homocharge accumulation near the electrodes is weakened, which reduces the effective height of barriers at the electrode-polymer interface and causes a large quantities of charges to accumulate in the dielectric bulk. The aggravation of space charge injection and accumulation could also improve the energy of hot electrons and accelerate the formation of the chain scissions or microvoids in amorphous regions, resulting in the decrease of breakdown strength. That is the main reason for the decrease of breakdown strength.

## Conclusion

In this paper, the effects of graphene addition on the dc conductivity, breakdown strength and space charge behaviors of LDPE/graphene composites are investigated, and their trap level distributions are analyzed. It can be summarized that the composites with a filler content of 0.005 wt% have a lower dc conductivity, a higher breakdown strength and a much smaller amount of space charge accumulation than the neat LDPE, which presents that the transport of charge carriers is suppressed by the well-distributed graphene. The density of deep traps increases with increasing filler content from 0 to 0.005 wt%, and then decreases with a further increase of filler content to 0.1 wt%, which is similar with the electrical performance. Accordingly, a schematic model is proposed for illustrating the effect of the polymer-filler interaction zones on charge carrier transport. It is concluded that large quantities of deep carrier traps distributed in the bonded layer and bound layer could capture the charge carriers in the vicinity of electrodes, thus enhancing the electric field required for charge injection and reducing the space charge accumulation in the dielectric bulk. Additionally, the formation of hot electrons with high energies are suppressed, resulting in the improvement of breakdown strength. However, further increasing of the filler content may lead to the charge transport through the overlapping sites of loose layers, and even an occurrence of thermally-activated field-enhanced carriers hopping between neighbor fillers, thus enhancing the dc conductivity macroscopically and reducing the breakdown strength of NCs. In conclusion, the deep trap modulated charge carrier transport is benefit to reduce the dc conductivity, modify the space charge behaviors and enhance the breakdown strength, which is of great significance in improving electrical properties of polymer dielectrics.

## Methods

### Preparation of LDPE/graphene NCs

LDPE (DFDB-6005 NT) with density of 0.92 g/cm^3^ is supplied by Dow Chemical Company (USA). The graphene nanoplatelets with a diameter of 0.5~5 μm and a specific surface area of 1217 m^2^/g are purchased from Hengqiu Graphene Technology Co., LTD (China). The graphene with different filler content of 0.005 wt%, 0.01 wt%, 0.1 wt% and 0.5 wt% are mechanically mixed with LDPE at the processing temperature of 453 K. To achieve proper dispersion of nanofillers in polymer matrix, the graphene is previously treated by surface modification agent. The mixed compounds are hot-pressed in a stainless steel mold at 433 K under a pressure of 15 MPa for 15 minutes. Then the prepared films are cooled down to room temperature.

### SEM and DSC tests

The cross-sections of specimens with different filler content are observed by scanning electron microscopy (SEM, Hitachi S4800). The specimens for SEM observing are previously fractured in liquid nitrogen and sputtered with thin gold layer. Crystallisation and melting of the LDPE/graphene NCs were studied in a Mettler-Toledo differential scanning calorimeter (DSC). The specimens (4.0 ± 0.5 mg) were heated to 180 °C and kept at this temperature for 5 min in order to erase the previous thermal history, cooled to 20 °C, and finally heated to 180 °C at a scanning rate of 10 °C min^−1^ to record the crystal melting.

### Electrical experiments

DC conductive characteristics of specimens with a thickness of 240 ± 5 μm are measured at room temperature. The experiments follow a standard procedure using a three-electrode system, where the high-voltage electrode is a stainless steel cylindrical electrode with a diameter of 30 mm, and the main electrode is 25 mm in diameter, where the guard ring eliminated surface currents. The charging current in an electrical field of 10 kV/mm is measured by an electrometer (Keithley 6517B) for 2100 s. Then the conductivity (*σ*) can be obtained by the following equation.

5 $$\sigma =\frac{I}{E}\cdot \frac{4}{\pi {(d+g)}^{2}}$$

where *I* is the mean value of the charging current during the final 100 s. *E* is the strength of the electric field. *d* is the diameter of the main electrode and *g* is the gap between the main electrode and cylindrical electrode. It should be emphasized that for each value of conductivity, a new specimen is used for current measurement to ensure the accuracy.

DC breakdown tests are performed in insulating oil at room temperature by using two stainless steel electrodes with a diameter of 6 mm. In order to reduce the edge effect, a chamfer with a radius of 0.5 mm is designed. The specimens with a thickness of 75 μm are tested at a voltage rising rate of 500 V/s until the specimens are broken down. Each specimen is tested more than 10 times and the two-parameter Weibull distribution is employed to characterize the dc breakdown strength.

Space charge distributions are tested by pulsed electro-acoustic (PEA) tests under an electrical field of 50 kV/mm. The specimens with a thickness of 240 ± 5 μm and a diameter of 8 cm are sandwiched between an aluminum electrode with a diameter of 12 cm and a semiconductive polymer electrode with a diameter of 2 cm. The voltage-on test is performed for 30 minutes, and the voltage-off test continues for 15 minutes. The space charge behavior of each group is confirmed by repeating the tests for five times.

IDC tests are employed to obtain the trap level distribution of composites with different filler content. The specimen with a thickness of 240 ± 5 μm is placed between two gold-coated stainless steel electrodes with a diameter of 2 cm. The tested specimen is first polarized under 30 kV/mm at 323 K for 30 min, and then short-circuited for 50 min to release the polarization charges. The isothermal discharge current curve is recorded by an electrometer (Keithley 6517B).
